# Urban Green Infrastructure Monitoring Using Remote Sensing from Integrated Visible and Thermal Infrared Cameras Mounted on a Moving Vehicle

**DOI:** 10.3390/s21010295

**Published:** 2021-01-04

**Authors:** Sigfredo Fuentes, Eden Tongson, Claudia Gonzalez Viejo

**Affiliations:** Digital Agriculture, Food and Wine Sciences Group, Faculty of Veterinary and Agricultural Sciences, School of Agriculture and Food, Parkville, VIC 3010, Australia; eden.tongson@unimelb.edu.au (E.T.); cgonzalez2@unimelb.edu.au (C.G.V.)

**Keywords:** urban tree management, tree monitoring, computer vision, tree water stress index, leaf area index

## Abstract

Climate change forecasts higher temperatures in urban environments worsening the urban heat island effect (UHI). Green infrastructure (GI) in cities could reduce the UHI by regulating and reducing ambient temperatures. Forest cities (i.e., Melbourne, Australia) aimed for large-scale planting of trees to adapt to climate change in the next decade. Therefore, monitoring cities’ green infrastructure requires close assessment of growth and water status at the tree-by-tree resolution for its proper maintenance and needs to be automated and efficient. This project proposed a novel monitoring system using an integrated visible and infrared thermal camera mounted on top of moving vehicles. Automated computer vision algorithms were used to analyze data gathered at an Elm trees avenue in the city of Melbourne, Australia (*n* = 172 trees) to obtain tree growth in the form of effective leaf area index (*LAIe*) and tree water stress index (TWSI), among other parameters. Results showed the tree-by-tree variation of trees monitored (5.04 km) between 2016–2017. The growth and water stress parameters obtained were mapped using customized codes and corresponded with weather trends and urban management. The proposed urban tree monitoring system could be a useful tool for city planning and GI monitoring, which can graphically show the diurnal, spatial, and temporal patterns of change of *LAIe* and TWSI to monitor the effects of climate change on the GI of cities.

## 1. Introduction

Green infrastructure (GI) has become a priority in most cities worldwide and has been recognized as an essential element in urban planning and development. The urban green infrastructure, which includes natural vegetation, parks, street trees, green roofs, and small gardens, provides various benefits to the environment, community, and the economy. The GI of a city contributes valuable benefits such as regulation and reduction of temperature during heatwaves [1] through plant transpiration [2], while green roofing decreases albedo [3]. GI improves air quality [2] and reduces flood risk [4,5] and stormwater pollution [6], among other environmental benefits. Beyond improving the ecosystem, GI has been linked to improving people’s physical and mental health [1]. Within the major challenges in urban cities are extreme heat and the urban heat island effect (UHI). UHI mainly occurs within cities with a higher proportion of concrete in relation to their green infrastructure (GI) [7]. In these cities, the ambient temperature increase can be multiplied by a 1.4–15 factor depending on circumstances within and surrounding a particular city environment [8,9]. UHI may worsen in the future, corresponding to the predicted climate change.

The maintenance of GI in cities may pose a challenge for city councils due to the number and complexity of tree and plant species, especially in cities classified as forest cities, such as Melbourne, Australia [1,2]. The City of Melbourne established a goal as part of the Urban Forestry Strategy to plant 3000 trees per year as one of the primary strategies for the climate adaptation program. The city council aimed to increase canopy to 140,000 trees in 2040, twice the coverage of the existing number of trees that it manages at present [3]. Trained arborists maintain public trees through routine inspection and assessment, which can also be requested through public reporting. Trees situated in heavily accessed areas such as parks and boulevards are inspected annually, while other locations are inspected at least once in 2 years. The city eliminates about 800 tree stands due to various reasons, including threats to safety. A considerable population of trees managed by the City of Melbourne are a century old and may pose higher risk of decline and, consequently, safety risk.

The manual inspection of urban trees by arborists is quite time demanding and inefficient. With the large-scale greening plans in urban cities such as Melbourne, it is highly impractical and nearly impossible to monitor GI to achieve high temporal and spatial resolution through the current manual practice. Other methods use wireless sensor networks, including the monitoring of soil moisture, temperature, light, humidity, and pressure [10], using internet of things (IoT) systems [4], real-time controls [5], and plant/tree-based sensors, such as sap flow probes [6,11]. These methods produce accurate and high temporal data resolution. However, one major disadvantage is that this method can only be applied to a few representative plants, as installing sensors to numerous trees is costly. Furthermore, the sensor networks require frequent monitoring and high maintenance, requiring specialized personnel with specific technical skills.

More spatially representative approaches for GI monitoring of cities are based on satellite remote sensing on relevant vegetation indices (VIs) related to growth and water status [12]. This has been applied in China for 70 major cities [13], and in Sweden [14] using Sentinel-2 and Landsat-8 satellites [13], as well as in Croatia using World View 1, 2, and 3 with high resolution visible and multispectral bands [15]. The use of satellite imagery can monitor large areas from a single image or stitched up images incorporating several square kilometers, which is a major advantage. Also, information can be readily available, and sources can be either free or low-cost (i.e., Landsat and Sentinel satellites). However, disadvantages can include low resolution of information per pixel, reaching 0.5 m for panchromatic imagery, and between 2 m to 30 m per pixel for multispectral imagery. Higher spatial resolution imagery may be expensive, such as those from the World View satellites. Furthermore, satellite revisit time to the same spot (i.e., in cities) may be between 10–15 days, and data quality depends on how clear the skies are.

To address the problem of low temporal and spatial resolution, airborne, and unmanned aerial vehicles (UAV) have been implemented to monitor GI in cities [16,17,18,19]. However, the use of airborne remote sensing comes with a cost, requiring a pilot and skilled personnel to operate the instrumentation, process the information, and deliver interpreted information to relevant city council personnel for GI management and decision making. Some services such as Nearmap (Nearmap, Barangaroo, NSW, Australia) offer high-resolution visible images with a high temporal resolution for major cities and coastlines [20,21]. However, the application of visible images is mainly for monitoring of growth parameters for trees [22]. The recent popularity of UAV has also expanded its application in remote sensing, with the accommodation of various camera and sensor payloads aside from visible such as multispectral camera and LIDAR [23,24,25,26]. With UAV, the main challenge is the implementation in countries with strict civil aviation regulations, such as many cities in Europe, the United States, and Australia, among others. In Australia, for example, the Civil Aviation Safety Authority (CASA) has a very strict regulation to fly drones within 30 m in proximity to people, making flights in heavily populated cities, such as Melbourne (Victoria, Australia) virtually impossible [27].

This paper proposes a novel GI monitoring approach based on prototype integrated visible and infrared thermal cameras to automatically obtain different VIs based on growth and tree water status parameters on a tree-by-tree scale. The integrated cameras are mounted on top of moving vehicles, circulated through one of the most important and historical Elm tree avenues in Melbourne (Royal Parade), Australia. The integrated system, which is composed of low-cost instrumentation, could be mounted on top of public transport vehicles, such as buses and trams, city council vehicles, and rubbish trucks. Public transport vehicles allow for incursion to potentially every street at multiple times in a day, offering a high temporal and spatial resolution of trees monitoring. The proposed system enables automated data acquisition, analysis, and mapping and does not require specialized personnel. It can also offer diurnal monitoring of trees to assess in real-time the effects of weather anomalies, such as heatwaves, floods, and heavy winds, among others, and the detection of pest and disease incidence. The novel technology (integrated cameras) and application could potentially be an accurate, cost-effective, and user-friendly tool for city councils, and they could base management strategies with high reliability on the system proposed, such as tree lopping, detection of encroachment of tree branches on power lines, and deterioration of old trees.

Only a few studies are based on the implementation of cameras for upward-looking imagery and analysis without any automation. These have been restricted to the 3D modeling of trees using handheld cameras and point cloud analysis [28] and the analysis of trees’ thermal characteristics [29]. Other applications using different technologies, such as hyperspectral cameras [30] and low-cost electronic noses (e-noses) [31], using the methodology proposed can be implemented to obtain more information from trees and their environment, such as diagnosis of vegetation health.

This study has been based on the integration of previously developed technology from our research group for the automated analysis of visible and infrared thermal imagery for different crops such as eucalyptus trees [22], grapevines [32,33,34,35], kiwi plants [36], apple trees [37], cherry trees [38,39,40], and cocoa plants [41], among others.

## 2. Materials and Methods

Data acquisition was performed in Melbourne, Australia, mainly based on an integrated visible and infrared thermal camera developed and processed using customized computer vision algorithms.

### 2.1. Urban Site and Tree Material Description

**Figure 1:**
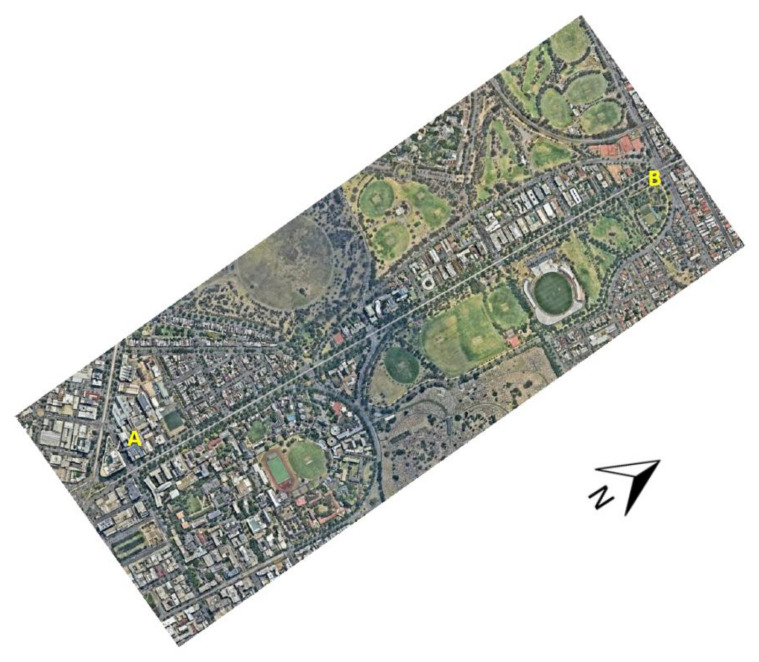
Location monitored using a moving vehicle from (**A**) start of Royal Parade from Grattan Street to (**B**) Park Street (2.52 km), and vice versa. There were 172 trees monitored, consisting of different species of Elm trees (*Ulmus* spp.).

The monitoring site (Figure 1) was located along the iconic Royal Parade avenue in the city of Melbourne, Australia, that starts at Grattan Street (−37°48′02.27″ S; 144°57′26.27″ E; 33 m.a.s.l.) finishing on Park Street (−37°46′41.45″ S; 144°57′36.56″ E; 46 m.a.s.l.), and vice versa. The trees are planted along a nature strip separating the main road and the access road in both directions. The main roads (North and South bound) are divided by a median strip containing the Route 19 tram lane. Each way corresponds to 2.52 km, with a total distance of 5.04 km both ways. There are 172 deciduous trees considered in the monitoring, composed of different Elm species (*Ulmus* spp.) planted in 1900 and 1997 [42]. The trees are irrigated using sub-surface irrigation, and tree lopping management is performed regularly by the Melbourne city council.

### 2.2. Climate and Weather Information Description

The climate in Melbourne is classified as subtropical oceanic with mild winters and pleasant to hot summers. Windy conditions are common, and weather changes can occur within the same day. The average temperatures between November and January are between 22 and 26 °C, with minimum temperatures between 11 and 14 °C. The yearly average precipitation is 670 mm, with an even distribution throughout the year of around 50 mm per month. Sunshine hours are higher between September and March (between 6–9 h). Specific weather data available for the trial site and the monitoring period were acquired from the Bureau of Meteorology, measured from a meteorological station located in Melbourne Olympic Park (Number: 086338) at 3.4 km from Royal Parade. The weather information extracted from this station was: maximum daily temperature (°C), rain (mm), and solar radiation (MJ m^−2^). Monitoring was performed from November 2016 (late spring) to January 2017 (summer).

### 2.3. Integrated Visible and Thermal Infrared Camera System

**Figure 2:**
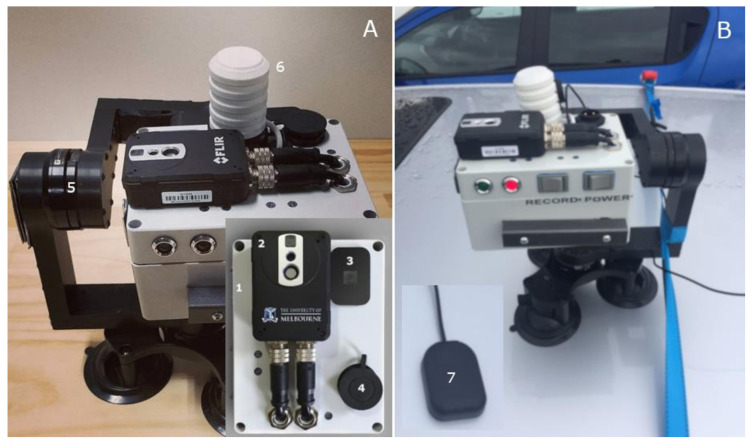
The integrated camera system. (**A**) The system is composed of weather and shock resistant case (1) that holds the Raspberry Pi boards and battery; thermal infrared camera FLIR AX8™ (2); the visible Red, Green, and Blue (RGB) Raspberry Pi Camera Module V2.1 (3); power mount receptacle (4) to charge the internal battery; 3-axis gimbal (5) to provide stability to the camera; integrated temperature, relative humidity, and solar radiation sensors (Stevenson screen, 6). (**B**) Example of the mounting procedure of the integrated camera on top of a vehicle. The camera was also integrated with a magnetic GPS tracker (7).

The integrated camera system (Figure 2) consisted of a visible RGB video camera and a thermal infrared camera FLIR AX8™ (FLIR Systems, Wilsonville, OR., USA) with a resolution of 90 × 60 pixels, connected to a web-based system that can simultaneously capture and store the videos and infrared thermal images (IRTIs) to be further downloaded for analysis or transmitted to cloud storage and processing system. The thermal camera had a spectral range of 7.5–13 μm, an accuracy of ±2 °C, and an emissivity of 0.985. The IRTI capture rate was every second. The RGB video camera is connected to a Raspberry Pi Camera Module V2.1 (Raspberry Pi Foundation, Cambridge, UK; Figure 2A), board, and memory card. This device has an 8-megapixel sensor with a resolution of 640 × 360 pixels, 4:3 aspect ratio, and 30 frames per second (fps). Videos were recorded within the unit in H.264 video compression format and automatically converted into Motion Pictures Expert Group-4 (.mp4) files. The camera was fitted with a 3-axis gimbal to minimize movements when acquiring the data (Figure 2A); an integrated temperature, relative humidity, and solar radiation sensors within a 3D printed Stevenson screen (Figure 2A); and a magnetic GPS tracker (Figure 2B). The integrated camera was mounted on top of a car (Figure 2B) with a height between the camera and the tree canopies of approximately 5 m.

The radiometric data from the thermal infrared camera were obtained every second while traveling through the Royal Parade. They were recorded as in comma-separated values (.csv) format files and the visible RGB images in Joint Photographic Experts Group (.jpg). Both sets of data were obtained using the Sense Batch software (SENSE Software, Warszawa, Mazowsze, Poland). The data were analyzed using customized codes developed and updated using Matlab^®^ R2020b (Mathworks Inc., Natick, MA, USA).

The camera’s integrated sensors consisted of an AM2302 (wired DHT22) temperature-humidity sensor (Guangzhou Aosong Electronics Co., Ltd., Guangzhou, China). This sensor can obtain new data from it once every 2 s (0.5 Hz), which is accurate for 0–100% humidity readings with 2–5% accuracy and −40 to 80 °C temperature readings with ±0.5 °C accuracy. The SP-510-SS upward-Looking Thermopile Pyranometer (Apogee Instruments, Inc., Logan, UT, USA) has a sensitivity of 0.05 mV per W m^−2^, with a measurement range between 0 to 2000 W m^−2^ (net shortwave irradiance) and repeatability of <1%. The detector response time is 0.5 s with a field of view of 180° and spectral range of 385–2105 nm, directional (Cosine) response less than 30 W m^−2^ at 80° solar zenith, temperature response: <5% from −15 to 45 °C at the operating environment: −50 to 80 °C, and 0 to 100% relative humidity.

### 2.4. Image Pre-Processing and Computer Vision Algorithms

**Figure 3:**
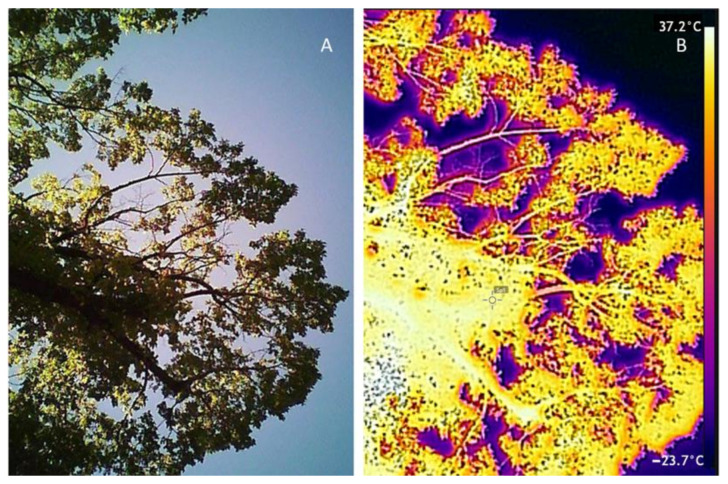
Example of a visible (RGB) image (**A**) and the corresponding infrared thermal image (**B**) from an Elm tree taken using the integrated camera on top of a moving vehicle.

Every frame corresponding to a canopy from the visible (RGB) video and infrared thermal images were analyzed using the computer vision algorithms described in Section 2.4.1 and Section 2.4.2, respectively. Figure 3 shows an example of a visible (RGB) frame and corresponding infrared thermal image from an Elm tree canopy along the Royal Parade.

**Figure 4:**
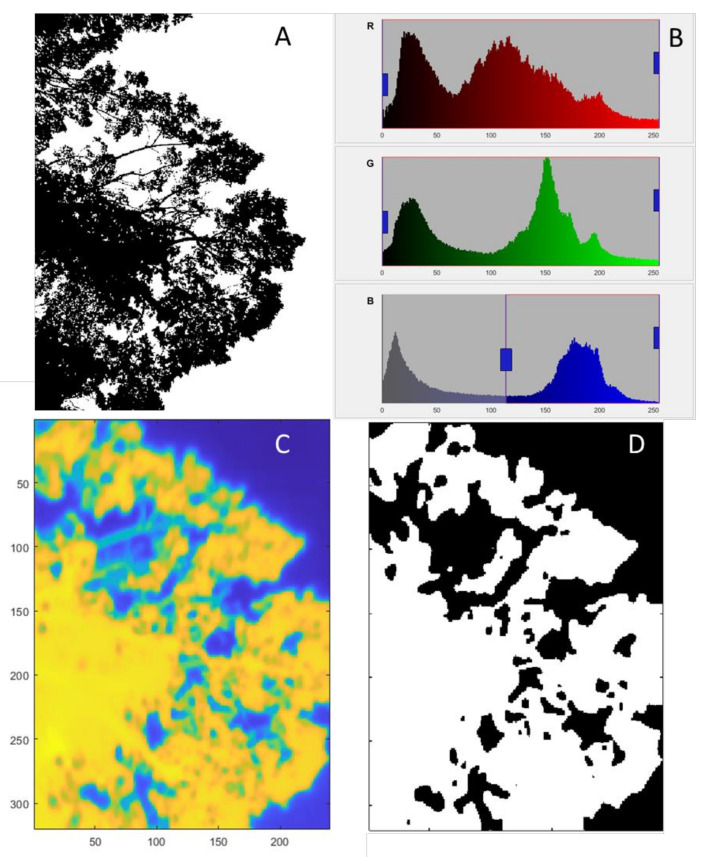
Example of the automated pre-processing of visible (RGB) images transformed to binary images (**A**) using the blue channel as filter (**B**) and the corresponding infrared thermal image (**C**) filtered to create a mask (**D**) to account for leaf material.

The pre-processing of the RGB images consisted of the binarization (Figure 4A) using the blue channel from the RGB images by selecting the lowest part of the histogram curve (valley) detected automatically between the pixels corresponding to the canopy material (first peak) and the background or sky (second peak) (Figure 4B). After binarization, each image was automatically subdivided into a 5 × 5 sub-images to perform gap analysis. A large gap (lg) per sub-image was considered when there was over 75% of sky. Total pixels (tp) corresponded to a fixed value related to the resolution of the camera used. This pre-analysis has been described in detail in Fuentes et al. [22].

The pre-processing of the thermal images was performed in batch after each measurement campaign using the SENSE Batch software (Sense Software, Warszawa, Mazowsze, Poland), which extracts radiometric data per pixel in a comma-separated file (.csv) in the form of a matrix processed in Matlab (Figure 4C). Leaf material was selected by simple automatic elimination of temperatures below 0 + °C since this separates the sky from the canopy material (Figure 4D). From the segmented image, the canopy temperature was automatically extracted (T_*canopy*_) as entry parameter for the TWSI and Ig calculation (Equations (7) and (8)).

#### 2.4.1. Canopy Architecture and Growth Parameters

Videos from the visible camera were processed automatically using a customized code written in Matlab^®^ R2020b to analyze frames following a computational process proposed by Fuentes et al. (2008) [22].

Canopy architecture parameters were obtained using the following algorithms considering the fractions of foliage projective cover (*f_f_*), crown cover (*f_c_*), and crown porosity (*Φ*), which were calculated using the following computational algorithms proposed by Fuentes et al. (2008) [22]:

(1) $$f_{f}=1-\frac{tg}{tp}$$

(2) $$f_{c}=1-\frac{lg}{tp}$$

(3) $$\phi =1-\frac{f_{f}}{f_{c}},$$

where *lg* = large gap pixels, *tg* = total pixels in all gaps, and *tp* = total gap pixels.

*LAI* (adimensional) is calculated from Beer’s Law, defined as the total one-sided area of leaf tissue per unit 3 ground surface area [43]. Hence, the *LAI* values describe m^2^ of leaf area per m^2^ of soil.

(4) $$LAI=-f_{c}\frac{\ln\phi }{k}$$

where *k* = coefficient of light extinction (*k* = 0.5), which is applicable for tall trees [22], and the clumping index at the zenith, Ω(0), was calculated as follows:

(5) $$\Omega \left(0\right)=\frac{\left(1-\phi \right)\ln\left(1-f_{f}\right)}{\ln\left(\phi \right)/f_{f}}.$$

The clumping index is a correction factor in obtaining effective *LAI* (*LAIe*), also adimensional, which is the product of:

(6) $$LAI_{e}=LAIx\Omega \left(0\right).$$

Equation (5) describes the non-random distribution of canopy elements. If Ω(0) = 1 means that the canopy displays random dispersion, then for Ω(0)> or <1, the canopy is defined as clumped.

#### 2.4.2. Infrared Thermal Image Analysis

A tree water stress index (TWSI) was derived from the common crop water stress index (CWSI) [32] used in agriculture, which is a normalized value (0–1) and, therefore adimensional, and it was calculated using the following equation after determining *T_dry_* and *T_wet_* [44]:

(7) $$T\mathrm{WSI}=\frac{T_{canopy}-T_{wet}}{T_{dry}-T_{wet}}$$

where *T_canopy_* is the actual canopy temperature extracted from the thermal image at determined positions, and *T_dry_* and *T_wet_* are the reference temperatures (in °C) obtained using the statistical temperature distribution discrimination described in published research [39].

An infrared index (*I_g_*), which is adimensional and proportional to leaf conductance and water vapor transfer (*g_s_*), can be obtained using the relationship as follows [45]:

(8) $$I_{g}=\frac{T_{canopy}-T_{wet}}{T_{dry}-T_{wet}}=\;g_{s}\;(r_{aw}+\left(\frac{s}{\gamma }\right){\;}_{r_{HR}})$$

where *r_aw_* = boundary layer resistance to water vapor, *γ* = psychrometric constant, and *s* = slope of the curve relating saturation vapor pressure to temperature [45,46].

For automated analysis, the leaf energy balance approached was implemented using integrated sensors within the camera described in Figure 2A as [32]:

(9) $$Tdry-Ta=\frac{r_{HR}R_{ni}}{\rho c_{p}}$$

where *Ta* is the air temperature measured at the same positions and time as infrared thermography acquisition, *r_RH_* = the parallel resistance to heat and radiative transfer, *R_ni_* is the net isothermal radiation (the net radiation that would be received by an equivalent surface at air temperature), ρ is the density of air, and *c_p_* is the specific heat capacity of air. This formula uses the concept of isothermal radiation and assumes a dry surface with the same aerodynamic and radiative properties, in which the sensible heat loss will equal the net radiation absorbed [47].

(10) $$Twet-Ta=\frac{r_{HR}r_{aW}\gamma R_{ni}}{\rho c_{p}\left[\gamma \left(r_{aW}\right)+sr_{HR}\right]}-\frac{r_{HR}\delta e}{\gamma \left(r_{aW}\right)+sr_{HR}}$$

The thresholds *Twet* and *Tdry* are references that can be leaves painted with water (*Twet*) and use petroleum jelly (*Tdry*) to obtain through infrared thermography the maximum and minimum temperatures to be found within a specific canopy at the time of measurements [32]. The leaf energy balance approach allows the implementation of an automated procedure to obtain these thresholds using the sensors incorporated in the integrated camera proposed (Figure 2).

### 2.5. Survey, Automated Detection of Trees Location, Data Extraction, and Mapping

Acquisition of images was performed on four dates: twice in November 2016 (17 and 19 November), followed by 19 December 2016 and 16 January 2017. The image surveys were all performed at 1–2 pm during maximum atmospheric demand (maximum vapor pressure deficit), a common practice in agriculture to assess plant water status for irrigation assessment requirements.

For the 172 Elm trees monitored in this study, the GPS location was extracted from Google Earth Pro (Googleplex, Mountain View, CA, USA). The tree positions were used as anchors to automatically extract information from procedures previously explained for canopy architecture and infrared thermal-based parameters. The automated extraction consisted of identifying the nearest coordinates registered in the integrated camera to the anchored GPS for specific trees.

Once the data were extracted, they were mapped using a customized code written in Matlab^®^ R2020b to produce: (i) geo-located icons (circles) with relative sizes to denote changes in growth (*LAIe*), and (ii) geo-located circles with a different color to represent different TWSI values. The process can be used to map any parameter extracted using Equations (1)–(10).

## 3. Results

### 3.1. Weather Data within the Period of Measurement and Calculated Parameters

**Figure 5:**
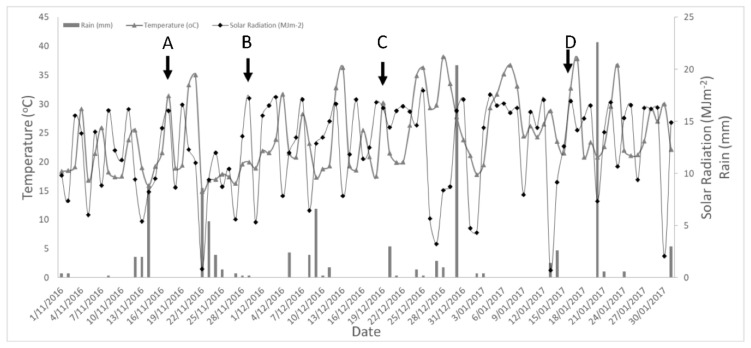
Meteorological data showing daily maximum temperature (°C) solar radiation (MJ m^−2^) and rain (mm) in the Royal Parade for four different dates studied: (**A**) 17 November 2016, (**B**) 29 November 2016, (**C**) 19 December 2016, and (**D**) 16 January 2017.

Figure 5 shows the weather information acquired from the closest meteorological station from the trial site. The first two dates of measurement (A: 17 November 2016 and B: 29 November 2016) had maximum rain events of 12.6 and 17 mm of rain in the previous week, and maximum temperatures of 31.4 and 20 °C, respectively. These dates had high solar radiation (28.8 MJ m^−2^ and 31 MJ m^−2^, respectively). In the last two measurement surveys (19 December 2016 and 16 January 2017), there were no or minimal rain events (0 mm and 4 mm, respectively) within two weeks preceding the measurements. Both dates had high maximum temperatures and solar radiation values (30.02 and 32.7 °C and 29.3 and 30.5 MJ m^−2^, respectively).

Table 1 shows the main canopy and tree water status parameters for all the measurement survey days. There was considerable variation in *LAI* and *LAIe* from a minimum of 0.61 and 0.41, found the last date of measurement, to maximum values of 5.98 for *LAI* in the first date of measurement and 4.97 *LAIe* for the second date of measurement. Furthermore, the lowest Tc values, TD, and TWSI corresponded to the second measurement date.

### 3.2. Comparative Analysis of Main Extracted Parameters from Trees

**Figure 6:**
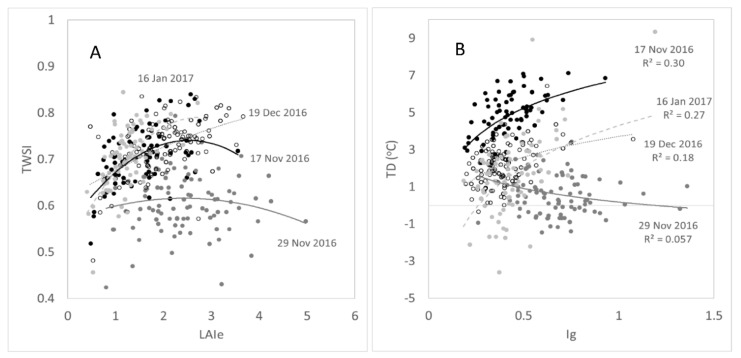
Comparison between effective leaf area index (*LAIe*, dimensionless) and tree water stress index (TWSI) (**A**), and between the infrared thermal index (*Ig*) and temperature depression (TD, °C) (**B**) for 172 elm trees monitored along the Royal Parade in Melbourne, Australia for four different dates between 2–16 and 2017, using the proposed urban tree monitoring system.

Figure 6A compares growth parameters (*LAIe*) for the 172 trees monitored with the TWSI for the different measurement dates. The trends followed apparent curvilinear relationships with the last two dates (19 December 2016 and 16 January 2017) with lower *LAIe* and higher TWSI than the earliest dates (17 November 2016 and 29 November 2016). Figure 6B shows the comparison between the Ig and TD parameters related to stomatal conductance from trees. There was contrasting behavior of these parameters for the first two dates with lower Ig and higher TD for the first and flat distribution of the whole range of Ig values with low TD close to the 0 values. On the contrary, the last two dates had similar behavior with low Ig and TD values ranging from −3 to around 3 °C.

### 3.3. Main Growth and Tree Water Stress Parameters Map

**Figure 7:**
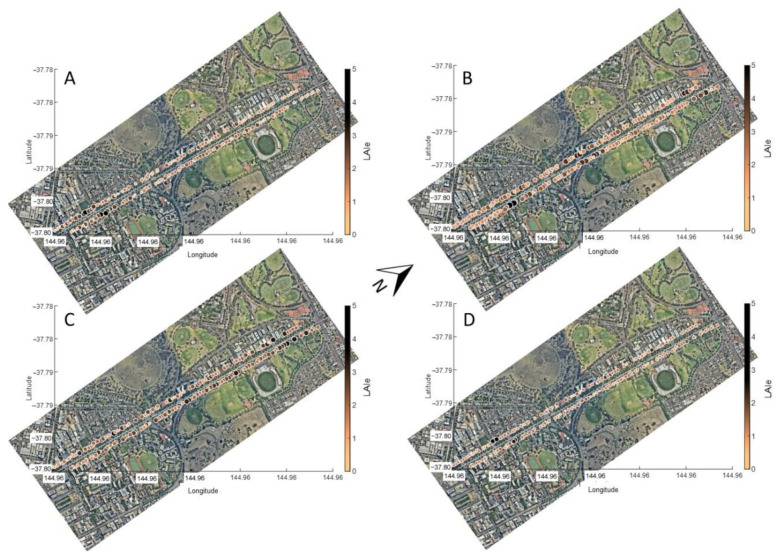
Mapping of effective leaf area index (*LAIe*) along the Royal Parade (5.04 Km) of 172 trees using the proposed urban tree monitoring system for four different dates: (**A**) 17 November 2016, (**B**) 29 November 2016, (**C**) 19 December 2016, and (**D**) 16 January 2017. Different colors and relative circle sizes correspond to the *LAIe* scale.

**Figure 8:**
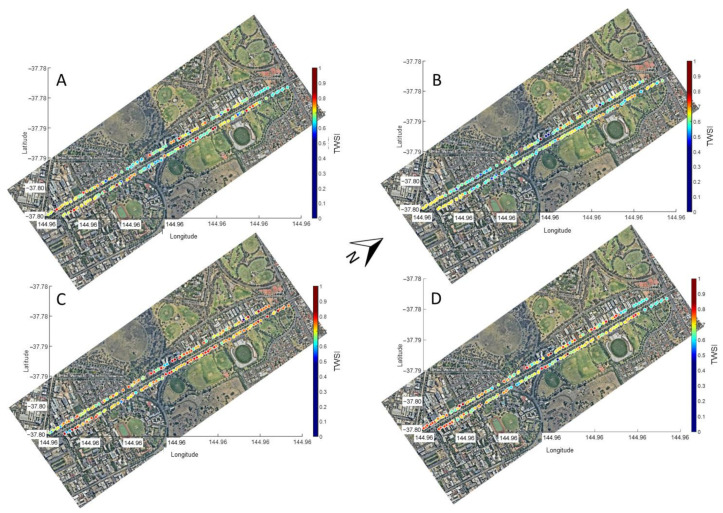
Mapping of tree water stress index (TWSI) along the Royal Parade (5.04 Km) of 172 trees using the proposed urban tree monitoring system for four different dates: (**A**) 17 November 2016, (**B**) 29 November 2016, (**C**) 19 December 2016, and (**D**) 16 January 2017. Different colors correspond to the TWSI scale.

Figure 7 and Figure 8 show the proposed urban tree monitoring system’s main outputs, displaying the main parameters extracted per tree along Royal Parade in four measurement dates. Figure 7 shows the *LAIe* for different trees with the relative size of circles corresponding to trees changing according to growth differences between dates. Figure 8 shows changes in color of circles representing the trees relative to the TWSI for different dates.

## 4. Discussion

The proposed urban tree monitoring system that uses an integrated camera on moving vehicles can automatically provide information on trees’ growth and water status changes, which can serve as a powerful decision-making tool for city councils for tree management (i.e., supply water requirement at appropriate times and tree lopping for power lines encroachment and public safety management). The reliability of the system is based on the growth and canopy architecture parameters and algorithms used, which have been successfully implemented for other trees such as eucalyptus [22], and tree crops such as cherry trees [38,40], apple trees [37], and grapevines [33,48,49,50]. Tree water stress algorithms have been used to describe the water status of many trees and crops [32,51,52,53]. Furthermore, most of the tree canopies were visible in the field of view of canopies for both visible and infrared thermal images (Figure 3 and Figure 4), making the analysis representative of the whole tree. Furthermore, since images are upward-looking, the monitored parts of the trees were the under canopy and were shaded, which has been regarded as the most consistent and representative part to monitor using infrared thermal imagery [46,54].

The sensitivity of the growth and physiological parameters obtained and their variations are specifically shown in Figure 6 and compared between the trees measured (individually) and temporally (within dates). The variation is sensible in response to weather conditions and changes related to atmospheric demand (temperature) and water availability (rain). These trends and their sensitivity are further supported by the mapping of the processed data in the form of *LAI* (Figure 7) and TWSI (Figure 8). The parameters obtained are in accordance with weather information acquired within the measurement dates. The first two dates (17 November 2016 and 29 November 2016) corresponded to milder weather, with cooler weather for the second date with a maximum temperature of 20 °C, followed by rain events. For the second date, lower atmospheric demands produced a flat response for TWSI and TD. In the case of TD, lower TD values, close to 0 °C, are related to low stomata opening and transpiration (Figure 6B). However, they were not associated with higher TWSI (Figure 6A). The rest of the dates (19 December 2016 and 16 January 2017) have more significant increases in TWSI with higher atmospheric demands (evapotranspiration), as shown by higher maximum temperatures and solar radiation. The highest and more significant determination coefficient (Figure 6B) between Ig and TD was found for the dates with higher atmospheric demand (first, third, and fourth dates), which was expected since these parameters are related to stomata aperture [32,54].

Another advantage of the proposed system is that it allows the automatic mapping of data obtained from surveys on a tree-per-tree scale (Figure 7 and Figure 8). For growth parameters, such as *LAIe* (Figure 7), some of the trees with higher growth showed decreased *LAIe* from the first to the second date of measurement, which may be related to continuous tree lopping management from the council (Figure 7A,B). However, the lowest and most consistent *LAIe* values were found in the last date of measurement (January 2017), which corresponded to one of the hottest months in summer and the starting of the senescence stage for the Elm trees (Figure 7D), in comparison to the previous dates. For TWSI, the parameter trends followed water availability from rain events during the last weeks and maximum temperatures with the highest values corresponding to the warmest dates in December 2016 and January 2017 (Figure 8C,D).

The integrated cameras could be mounted on public transport of cities, such as buses and trams. The installation of the system on trams is ideal, being on rails are on a fixed route, which can offer more precise data acquisition and more reliable comparative analysis. Furthermore, at least along the Royal Parade route (Route 19), a particular tram can pass through the same spot every 80–90 min, which can acquire at least 13 data points in one day from 5 am to midnight. To access more places within the city, such as suburb streets, cameras could be installed on rubbish trucks and buses, which have more extensive access to residential areas. This layout of the trams’ path is similar to many European cities since they have similar designs.

The diurnal data collected may be more relevant for infrared thermal parameters to assess tree water status changes throughout the day compared to changes in growth, which are expected to be minimal, while data related to changes in leaf or branch angle due to water stress after sunset could be relevant to assess night-time water loss by trees, as this phenomenon is relevant to other tree species and crops [55,56,57,58,59]. Continuous daily data of water stress may also offer insights of tree behavior within heat waves [60], pest and disease interactions [61,62], windy days, and mortality estimates [63]. The volume of data that can be gathered through the proposed system allows the implementation of machine learning modeling and artificial intelligence to promptly detect problems for management and mitigation, avoiding damage to infrastructure and the public due to unpredicted fallen trees or big branches.

The system has been proposed, and data analysis can be deployed as a user-friendly digital platform producing maps with tree water status and growth maps depicted in Figure 7 and Figure 8. Users can click on any individual tree and obtain numerical and other management information as it is already set up for planting date and basic information of trees by the Melbourne city council [3]. Furthermore, since the conception of the integrated camera idea, on which this paper was based, FLIR has released an integrated visible 4K video camera and a high resolution infrared thermal imaging: FLIR Duo Pro (FLIR Systems, Wilsonville, OR, USA). This camera is intended to be mounted as a payload for UAV vehicles. It can also be used to acquire data to obtain the analysis proposed in this paper mounted on vehicles as per Figure 2B. The downside will be the costs of using these cameras if many vehicles are required for this purpose. It is thought that the higher resolution from the FLIR camera will not impact with statistical significance results obtained with the low-cost camera system presented in this paper. The latter is supported by previous research that has compared different resolutions of visible and thermal infrared cameras for growth and water status assessment on trees with no significant differences for the parameters studied [39,64], which can be explained by the short height between the camera and the canopies included for these type of studies, which is between 3–5 m.

This study was based on an extensive avenue in which there were a predominant tree species. Hence, further studies should be conducted for different tree species to account for the variety that exists in a normal urban green infrastructure environment. Even though the algorithms used in this study have been proven to be robust for other horticultural tree species, specific calibrations should be made to consider different canopy architectures and sensitivity/tolerance to different water stress levels.

## 5. Conclusions

The urban green infrastructure could be automatically monitored using a low-cost integrated camera system mounted on top of moving vehicles. Specifically, the main advantages of the system described in this paper compared to similar studies to monitor the green infrastructure in urban environments are: (i) low-cost instrumentation required to integrate visible and infrared thermal cameras; (ii) the system can be mounted on public transport such as buses, trams, and city council vehicles with the extra advantage when considering garbage trucks since they can access every street of a city if extensive monitoring is required; (iii) it could provide high spatial and temporal data resolution, which is related to the frequency of public transport through the same trees; (iv) algorithms implemented are robust and have been successfully tested on a wide variety of horticultural trees; (v) the system does not require special permits or trained pilots, such as the case of UAVs, and they also do not have restrictions due to privacy issues since they monitor urban infrastructure in an upward-looking fashion above the pedestrian level. These operational, cost-effectiveness, accuracy, and privacy-related advantages of the system proposed can be compared to those of manual measurements of green infrastructure, using sensors and IoT on sentinel trees, remote sensing using satellites, UAVs, or the airborne instrumentation (Nearmap) discussed in this paper. Furthermore, the high volume of data collected (spatial and temporal) using the system proposed in this paper could allow the implementation of machine learning algorithms and artificial intelligence (AI) to obtain further vegetation indices of trees to manage the cities’ green infrastructure efficiently, to maximize resources, and to minimize detrimental effects of climate change and risk to infrastructure and people.

## Figures and Tables

**Table 1:** Growth and water stress parameters obtained using the proposed urban tree monitoring system, measured at Royal Parade in Melbourne, Australia, for four measurement surveys between 2016 and 2017. Parameters are presented with maximum, minimum, means, and standard deviation values (SD) for leaf area index (*LAI*, adimensional), effective *LAI* (*LAIe*, adimensional), canopy temperature of trees (Tc, °C), temperature depression (TD, °C), thermal infrared index (*Ig*, adimensional), and tree water stress index (TWSI, adimensional).

Parameter/Date	17 November 2016				29 November 2016				19 December 2016				16 January 2017			
	Min	Max	Mean	SD	Min	Max	Mean	SD	Min	Max	Mean	SD	Min	Max	Mean	SD
*LAI*	0.81	5.98	2.67	±1.17	0.86	5.33	2.58	±0.90	0.63	4.88	2.70	±0.88	0.61	4.11	1.86	±0.57
*LAIe*	0.48	3.56	1.59	±0.70	0.80	4.97	2.41	±0.84	0.47	3.67	2.03	±0.66	0.41	2.72	1.23	±0.38
Tc	25.9	30.7	28.2	±1.21	16.5	21.5	19.3	±1.10	23.6	30.3	27.9	±1.05	23.7	36.6	31.5	±1.99
TD	0.7	5.5	3.2	±1.21	−1.5	3.4	0.7	±1.10	−0.1	6.6	2.3	±1.05	−3.9	9.1	1.3	±1.99
*Ig*	0.19	0.93	0.43	±0.12	0.26	1.36	0.66	±0.17	0.20	1.07	0.39	±0.12	0.18	1.19	0.45	±0.15
TWSI	0.52	0.84	0.70	±0.06	0.42	0.79	0.61	±0.06	0.48	0.84	0.73	±0.06	0.46	0.84	0.70	±0.07

## Data Availability

Data and intellectual property belong to The University of Melbourne; any sharing needs to be evaluated and approved by the University.
